# Supplementary material for: Neosaxitoxin Inhibits the Expression of Inflammation Markers of the M1 Phenotype in Macrophages
Source: Mar Drugs. 2020 May 27;18(6):283. doi: 10.3390/md18060283 (PMC7345530; doi:10.3390/md18060283)
Supplement: Supplementary file 1 [file marinedrugs-18-00283-s001.pdf]

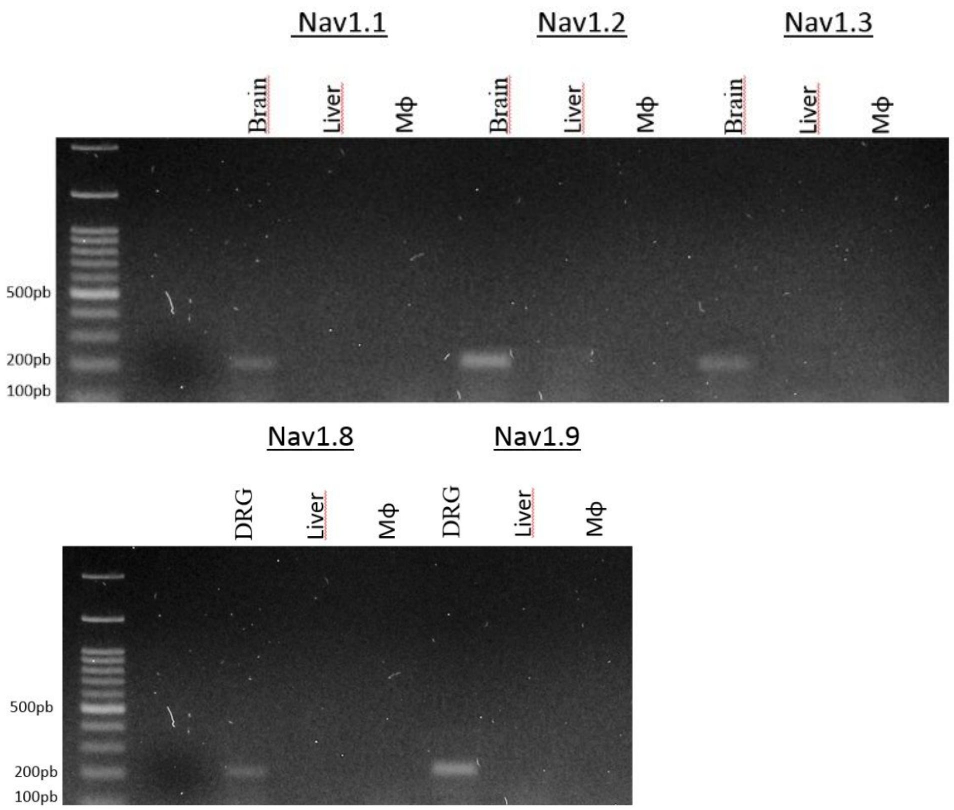

Figure S1.- Figure 2. Characterization of the Nav the Isoforms in a macrophage model. (A) Expression of Navs in primary culture. RT-PCR product for isoforms of Nav 1.1, 1.2, 1.3, 1.8 and 1.9 in equine PBMC (Mϕ). Positive control (Brain and Dorsal Root Ganglion (DRG)) and negative control (Liver) are shown.

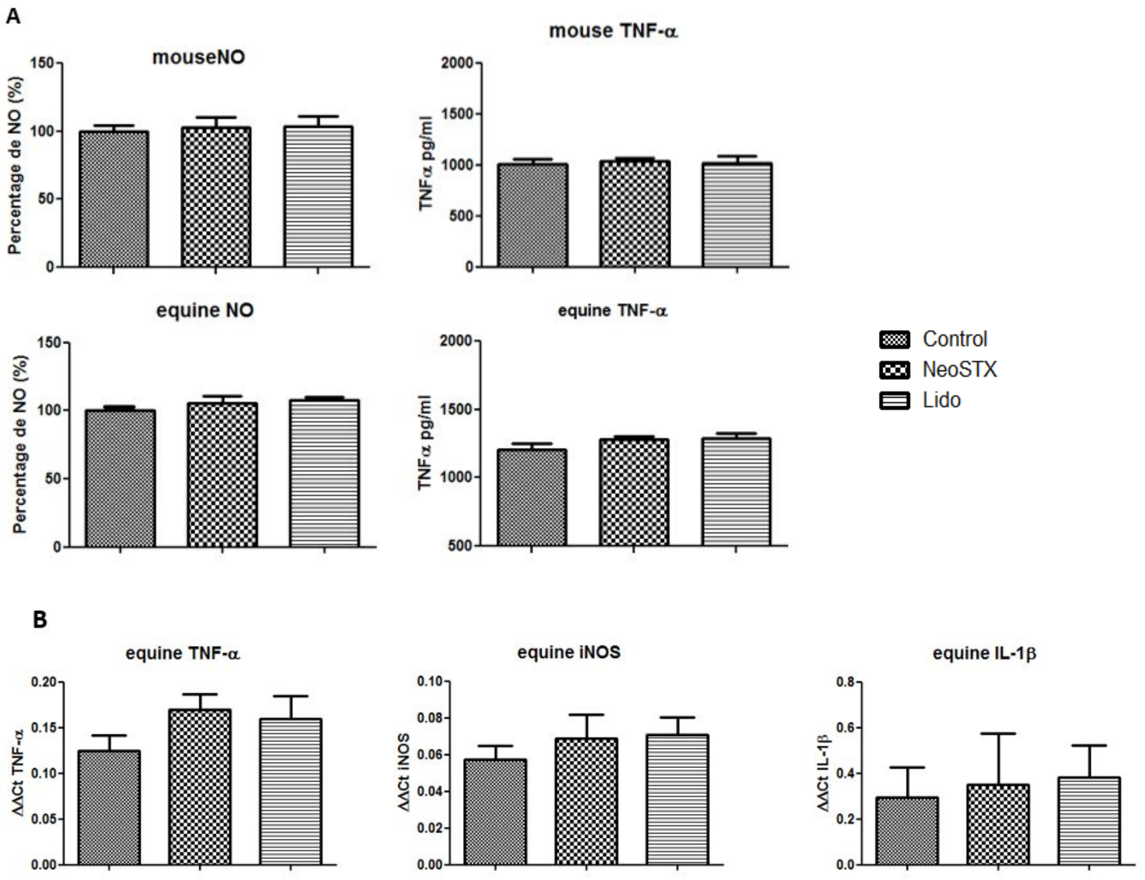

Figure S2.- Effect of NeoSTX and Lidocaine on RAW 264.7 cells and primary culture. (A) Cells were cultured in the presence of NeoSTX (1μM) or lidocaine (20μg/ml) for 24 hours. The production of NO and TNF-α in the culture supernatant was quantified using the Griess technique and ELISA technique respectively. (B) The expression of the equineTNF-α, equineiNOS, and equineIL-1β mRNA was quantified by RT-PCR (C). β-Actin was used as a control gene. * $p < 0.05$ (n = 4).

Table 1.- Primers for the Nav isoforms (1.1 to 1.9) described for the equine species.

PREDICTED: Equus caballus sodium channel	Forward	Reverse	Product Length (pb)
Nav 1.1 (SCN1A) XM_001916693.4	TGGCTATGGCCTATGAGGAACAGA	GAACTCAACTTGGAGGCTTCCGAT	208
Nav 1.2 (SCN2A) XM_014732515.1	GACTGTCCCAATTGCTGTTGGAGA	TGTGAAACAGGCTTCAGGTTCGAG	208
Nav 1.3 (SCN3A) XM_014732264.1	CTCTAATCGGTCTGCAGCTGTTCA	TCGTGTAGCCATAGTTGGGGTTTC	204
Nav 1.4 (SCN4A) XM_005597271.2	TGAGCAAGATGTATGGCCGTGAGA	TTGATGCAGCCTGCTAGACAAGAG	201
Nav 1.5 (SCN5A) NM_001163895.1	ATGTGCACCATCCTGACCAACT	TGCCATGACAATCACGCTGAAG	203
Nav 1.6 (SCN7A) XM_014732516.1	AAGACTGTCAACTCCCACGATG	GCTCACCAATGCCAGAAACAGGTA	210
Nav 1.8 (SCN8A) XM_014740776.1	TTCCGATTGCTCCGAGTCTTCA	AGGGAGTTCACAGTCCTGGTTGAT	210
Nav 1.9 (SCN9A) XM_001496423.4	TCACAGTGACAGTGCCAATTGC	GCCTCTGGCTCATCTGAATTCACA	205
